# Case study of medical evacuation before and after the Fukushima Daiichi nuclear power plant accident in the great east Japan earthquake

**DOI:** 10.1186/s40696-015-0009-9

**Published:** 2015-10-30

**Authors:** Tetsu Okumura, Shinichi Tokuno

**Affiliations:** 1 Countermeasures against NBC (Nuclear, Biological, and Chemical) Threats, Office of Assistant Chief Cabinet Secretary for National Security and Crisis Management, Cabinet Secretariat, Government of Japan, Nagatacho 2-4-12, Chiyoda, Tokyo 100-0014 Japan; 2 Verbal Analysis of Pathophysiology, Graduate School of Medicine, The University of Tokyo, Tokyo, Japan

**Keywords:** Disaster, Medical transportation, Great East Japan earthquake, Government

## Abstract

**Introduction:**

In Japan, participants in the disaster-specific medical transportation system have received ongoing training since 2002, incorporating lessons learned from the Great Hanshin Earthquake. The Great East Japan Earthquake occurred on March 11, 2011, and the very first disaster-specific medical transport was performed. This article reviews in detail the central government’s control and coordination of the disaster medical transportation process following the Great East Japan Earthquake and the Fukushima Daiichi Nuclear Power Plant Accident.

**Case description:**

In total, 124 patients were air transported under the coordination of the C5 team in the emergency response headquarter of the Japanese Government. C5 includes experts from the Cabinet Office, Cabinet Secretariat, Fire Defense Agency, Ministry of Health, Labour and Welfare, and Ministry of Defense. In the 20–30 km evacuation zone around the Fukushima Daiichi nuclear power plant, 509 bedridden patients were successfully evacuated without any fatalities during transportation.

**Discussion and evaluation:**

Many lessons have been learned in disaster-specific medical transportation. The national government, local government, police, and fire agencies have made significant progress in their mutual communication and collaboration.

**Results:**

Fortunately, hospital evacuation from the 20–30 km area was successfully performed with the aid of local emergency physicians and Disaster Medical Assistance Teams (DMATs) who have vast experience in patient transport in the course of day-to-day activities. The emergency procedures that are required during crises are an extension of basic daily procedures that are performed by emergency medical staff and first responders, such as fire fighters, emergency medical technicians, or police officers. Medical facilities including nursing homes should have a plan for long-distance (over 100 km) evacuation, and the plan should be routinely reevaluated with full-scale exercises. In addition, hospital evacuation in disaster settings should be supervised by emergency physicians and be handled by disaster specialists who are accustomed to patient transportation on a daily basis.

## Background

In Japan since 2002, participants in the disaster-specific medical transportation system, which include staff of local hospitals, emergency department staff, emergency medical technicians, police and fire fighters, are consistently trained in exercises for disaster-specific medical transportation. On March 11, 2011 at 14:46, the Great East Japan Earthquake hit the northeastern part of mainland Japan. It was the most powerful earthquake ever recorded to hit Japan, and the earthquake triggered powerful tsunami waves that reached heights up to 40.5 m. The National Police Agency confirmed 15,891 dead, 6152 injured, and 2584 missing. When this earthquake occurred, the very first disaster-specific medical transport in Japan was initiated [1]. This article reviews, in detail, the central government’s coordination of the disaster medical transportation process following the Great East Japan Earthquake and the Fukushima Daiichi Nuclear Power Plant Accident, and includes the roles and actions of the central operation and the lessons learned from this disaster. Shimada et al. reported on the hospital evacuation following the Fukushima nuclear accident from the viewpoint of local emergency physicians [2]. In this article, we present the government’s coordinating role from the viewpoint of central government administrative officers and lessons learned from the viewpoint of governmental disaster management. Recommendations for future efforts are discussed.

On the morning of January 17, 1995, the Great Hanshin (Osaka-Kobe) Earthquake resulted in 6308 deaths and 35,000 injuries [3]. In Japan, before the Great Hanshin Earthquake, disaster medicine and the concept of disaster-specific medical transportation were not well established. In fact, on the day of the Great Hanshin Earthquake, only one trauma patient was transported by helicopter from the devastated area to an outside hospital. In the first 3 days after the Great Hanshin Earthquake, only nine patients were transported by helicopters. Many critically injured patients were left in the devastated area without appropriate trauma care, at least 500 deaths due to trauma caused by this disaster were thought to be preventable.

In 2000, the Doctor-Heli system was introduced [4]. The Doctor-Heli system comprises a helicopter that carries an emergency trauma physician and a nurse with emergency medical rescue equipment to the scene of a disaster. This prefecture- and government-funded helicopter system enables the medical team to begin emergency treatment on-site and during transport to a nearby trauma care facility. By 2012, 30 of the 47 prefectures in Japan had introduced the Doctor-Heli system.

In 2002, the South Kanto area Great Earthquake tabletop exercise was performed. In this exercise coordinated by the Cabinet Office of the Government of Japan, the main theme was medical transportation during a disaster situation. The Cabinet Secretariat, Cabinet Office, Ministry of Health, Labour and Welfare (MHLW), Ministry of Defense, and other related ministries made concerted and united efforts to communicate and collaborate to ensure medical transportation for victims who sustained injuries during an earthquake.

**Fig. 1:**
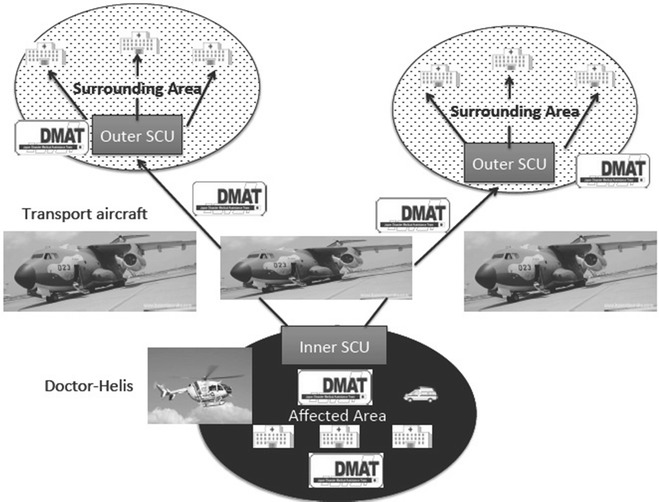
Initially, Disaster Medical Assistance Teams (DMATs) go from the outer staging care units to the inner staging care units. DMATs take patients from the inner staging care units to the outer staging care units. *Arrows* show patient flow.* JSDF* Japan Self-Defense Force

Systemization of medical transportation following a disaster started with the lessons learned from the Great Hanshin Earthquake, in which many persons were died without appropriate trauma care; a major contributing factor was that devastated hospitals were not able to provide appropriate trauma care. With these lessons learned, DMATs were introduced in 2004 throughout Japan. Education and central coordination of DMATs are overseen by the MHLW, and the operation and maintenance of DMATs are managed by each prefecture. Members of DMATs are emergency trauma physicians, nurses, pharmacists, and clerks. Following a disaster, the main purpose of the medical transportation system is to save the lives of trauma patients and to provide support for hospitals that are overwhelmed by casualties. For this purpose, DMATs are first gathered at hubs outside the disaster area (outer staging care units: outer SCUs) and then transported to affected hospitals via hubs inside the affected area (inner SCUs). Trauma patients are then transported to the outer SCUs via the inner SCUs (Fig. 1).

Medical transportation inside the disaster area is mainly done by helicopters, including Doctor-Helis and ambulances, and outside the disaster area by fixed-wing planes of the Self-Defense Forces. Critical patients are accompanied by DMATs who continue to care for patients during transport.

In 2004, at the time of the Niigata Chuetsu Earthquake, a Doctor-Heli was dispatched to the affected area; this was the very first disaster mission for the Japanese Doctor-Heli [5]. Since 2004, full-scale exercises of disaster-specific medical transportation are performed annually. Year by year, participants are becoming more adept and proficient.

The Cabinet Office has created a manual of the governmental emergency response headquarter (ER-HQ). According to this manual, ER-HQ is divided into three A teams (Integration Teams), three B teams (Information Teams), eight C teams (Operation Teams), and five D teams (General Affairs Team). The manual also describes the teams and roles that are required during disaster management (see Table 1). Members of the C5 team are from the Cabinet Office, Cabinet Secretariat, Fire Defense Agency, MHLW, and Ministry of Defense. They are tasked with the specialized coordination of disaster medical transportation. In 2007, the Cabinet Secretariat additionally invited an emergency physician to act as a government official and consultant.

**Table 1:** Disaster management teams and tasks in the ER-HQ of the Japanese Government

Group of teams	Team	Tasks
A: Integration teams	A1	Integration of real-time situations
	A2	Tactics building and analysis
	A3	Meeting and public relations
B: Information teams	B1	Integration of information
	B2	Information sharing
	B3	Reporting of collected information
C: Operation teams	C1	Integration of operations
	C2	Response
	C3	Transportation
	C4	Logistics
	C5	Medical transportation
	C6	Reception of international aid
	C7	Sheltering and evacuation
	C8	Infrastructure and life lines
D: General affairs	D1	Integration of general affairs
	D2	Distribution of food and nutritional supplements
	D3	Publicity
	D4	General affairs and communication
	D5	Dispatch of government persons

## Case description

### Early stage before the Fukushima Daiichi accident: the first challenge

**Fig. 2:**
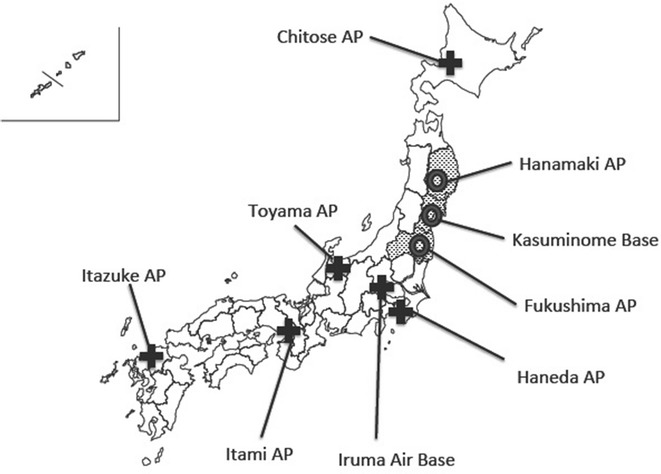
Inner staging care units and outer staging care units. The *broad cross* shows the outer staging care unit and the *circle* shows the inner staging care unit. *Dotted areas* are the three main earthquake-affected prefectures: Iwate, Miyagi, and Fukushima prefectures

Within the Japanese government, the practical business of disaster response is the responsibility of the Cabinet Office. Immediately after the Great East Japan Earthquake happened, the governmental ER-HQ was set up by the Cabinet Office headed by the Prime Minister. As planned, the C5 team was activated. The consulting emergency physician was dispatched by the Cabinet Secretariat to assist the C5 team. All members of the C5 team were experts in the field of medical transportation. The initial task of the C5 team was to facilitate the establishment of inner SCUs and outer SCUs (Fig. 2).

**Fig. 3:**
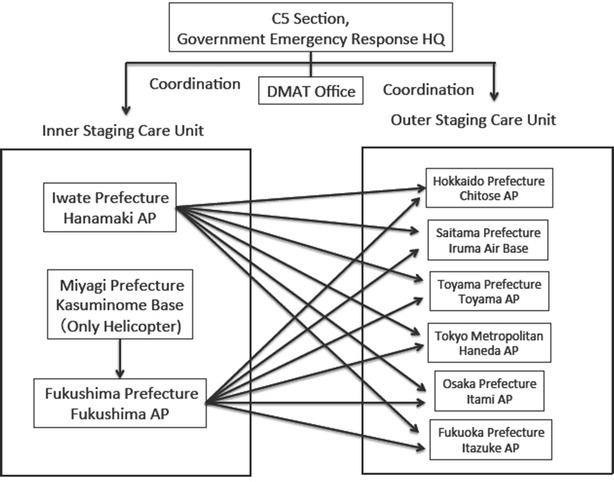
Flow of patient air transportation. The earthquake affected Iwate, Miyagi, and Fukushima prefectures. *DMAT* Disaster Medical Assistance Team,* HQ* headquarters and * AP* airport

On the night of March 12, transportation of victims to the outer SCUs had begun. Figure 3 shows the transportation flow. The Doctor-Heli system also played a remarkable role in medical transportation inside the affected area. In total, 16 Doctor-Helis came from unaffected areas and they treated and transported 149 critical patients [6, 7].

Disaster transportation was primarily handled by the prefectures. When local governments experienced difficulty in the coordination of disaster transportation, they could ask the central government for help as needed. Otherwise, the head office of DMATs or the MHLW requested coordination of disaster transportation. The C5 team received requests from any organization, including private sectors, and obtained as much information as possible. In total, 124 victims were transported by air (Table 2). This number does not include the evacuation from the Fukushima Daiichi nuclear power plant accident. Among all the evacuees we coordinated, 19 were trauma patients. In the Great East Japan Earthquake, the main cause of injuries was the tsunami; while many people died, survivors had few or relatively minor injuries and few had serious trauma injuries. On the other hand, the earthquake and tsunami damaged the hospital in the affected area and the demand for transport of patients on dialysis and patients with neuro-degenerative diseases who required artificial ventilation gradually increased over time; 94 patients transported on March 23 were dialysis patients. The last case transported was a patient who needed to return home (April 21 in Table 2).

**Table 2:** Number of patients air-transported with the coordination of the C5 section

	Plane and helicopter of the self-defense forces	Fire fighting helicopters	Daily account	Cumulative total value
March 11			0	0
March 12		5	5	5
March 13	11		11	16
March 14		3	3	19
March 15		2	2	21
March 16			0	21
March 17			0	21
March 18	4		4	25
March 19	4		4	29
March 20			0	0
March 21			0	0
March 22			0	0
March 23	94		94	123
March 24			0	0
March 25			0	0
Apr 21	1		1	124

Most of the patients included in the medical transportation scheme were treated in an unaffected area, recovered, and returned home using the usual transportation system. One particular patient stabilized, but required continuous treatment and could not return by surface transport. The patient and his family wanted to go back to the hospital in their hometown and requested air transport. Typically, return transportation cases are not included in the disaster transportation scheme; however, MHLW and the Ministry of Defense negotiated and the patient was finally transported by the Aero-Medical Evacuation Squadron (AMES) of the Japan Air Self-Defense Force. The AMES is a so-called flying intensive care unit. This case was the first for the AMES. The AMES uses C130H transport aircrafts with Aero-Medical Evacuation Units. The AMES may be used for medical evacuation in future disaster settings.

During the height of the chaos, the requests for transportation contained confusing information, and duplicated information was often received. For example, a prefecture asked to transport 400 dialysis patients, so ten buses were designated and prepared for this purpose. The same prefecture subsequently sent another request for the C5 team to transport 400 patients on dialysis, so the C5 team obtained an additional 10 buses, only to have both requests cancelled by the prefecture.

### After the Fukushima Daiichi accident: the second challenge

The tsunami damaged the Fukushima nuclear power plant, and day by day the situation at the power plant deteriorated. The most tragic situation in the early stage of medical transportation was at Futaba Hospital. Futaba Hospital (including Deauville Futaba, an adjunct nursing home to Futaba Hospital) was within 5 km of the Fukushima Daiichi nuclear power plant accident and patient evacuation was required. During the chaos, Futaba Hospital lost three patients before the morning of March 14, and a total of 50 patients by the end of March due to the inappropriate medical care circumstances [8]. In the process of the evacuation, Futaba Hospital patients were initially moved to a general evacuation center with healthy citizens and subsequently moved to hospitals. Each of the four hospitals in the 20-km zone should have had a concrete evacuation plan and conducted drills as part of a prefectural disaster plan, but in reality only one of these hospitals had an evacuation plan.

**Fig. 4:**
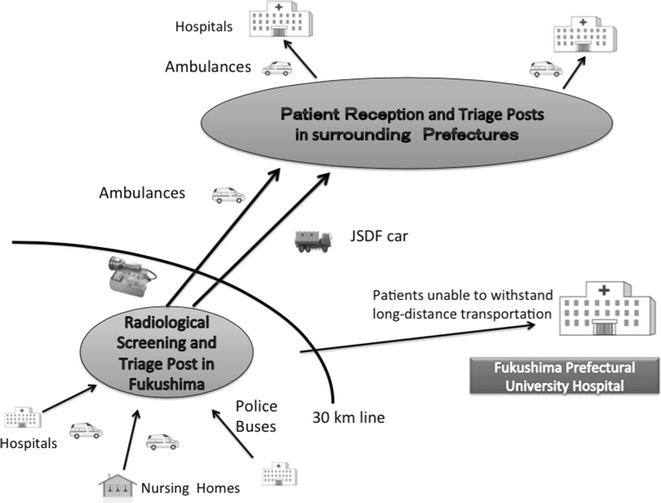
Evacuation scheme in the Fukushima nuclear evacuation

Finally on March 15, the government decided to define the evacuation zone as a 30-km circle around the nuclear power plant. At that time, there were 1000 bedridden patients in hospitals and nursing homes in the evacuation zone. These 1000 bedridden patients needed transport from the inner area to an outside area as soon as possible. This was a difficult task and absolutely beyond the capacity of the air transportation scheme. Meanwhile, the DMATs had started to withdraw out of Fukushima. Originally, the activity period of the DMATs was defined as the first 72 h after the disaster. The C5 team requested the DMATs to help with this task. In the early stage, real-time information on the medico-social situation of the evacuation zone was lacking; therefore, two members of the C5 team including an emergency physician were dispatched to the Fukushima governmental headquarters (HQ) on March 17. The mission of the two C5 delegates was to communicate, coordinate, and collaborate to save patients, including the 1000 bedridden patients in the 20–30 km evacuation zone. When the C5 team members reached the prefectural HQ, local government employees were trying to match patients from inside SCUs to outside SCUs one case at a time. This excessive coordination proved to be labor-intensive and ineffective. Thus, if the medical transportation team were to spend their time in this manner, they could never successfully evacuate a large number of patients. Therefore, the C5 delegation decided that the receiving prefecture must accept the entire group of patients, knowing only the number of patients and distribution of injury severity. The delegation negotiated with the concerned organizations that were part of the coalition of the central C5 team. Surrounding prefectures came to terms with this dynamic scheme under the strong leadership of the MHLW and finally the transportation started to proceed smoothly. Figure 4 shows this scheme.

In this scheme, bedridden patients were first collected at the screening and triage area. In theory, patients who had been in the hospital had no risk of radiation contamination. However, surrounding prefectures that had not sustained damage from earthquakes, the tsunami, or the nuclear accident were concerned about radioactive contamination on patients. At the 30-km line, radiation screening and triage were established, and DMAT and other teams checked the patients for contamination, evaluated the physical status of patients, and performed triage. According to the patients’ physical status, transportation measures (helicopters, ambulances, buses) were selected. Patients who were unable to withstand long-distance transportation were sent to the local central hospital, Fukushima Prefectural University Hospital. As a result, 509 patients were successfully transported from inside to outside of the evacuation zone without any deaths occurring during transportation. Voluntary non-governmental organizations were also engaged in transportation from the evacuation zone; unfortunately, several deaths were reported during the transportation, presumably because of inexperience in medical transportation.

In Japan, the Fire and Disaster Management Agency established a system to allow the central government to request municipal fire brigades to form emergency rescue teams in the event of large-scale disasters such as major earthquakes and accidents at nuclear power plants. This scheme was also supported by unaffected local governments from all over Japan. Ambulances are not fuel efficient and require large quantities of gasoline. The C5 team members, in close collaboration with the local branch of the Ministry of Economy, Trade, and Industry, were able to maintain sufficient quantities of gasoline. The C5 team also coordinated the transportation of medical oxygen cylinders and special drugs such as methylene blue, which were needed for the nuclear crisis.

## Discussion and evaluation

Many lessons have been learned in disaster-specific medical transportation. The national government, local government, police, and fire agencies have made significant progress in their mutual communication and collaboration.

Fortunately, hospital evacuation from the 20–30 km area was successfully performed with the aid of local emergency physicians and DMATs who have vast experience in patient transport in the course of day-to-day activities. Voluntary non-governmental organizations were also engaged in transportation with good intentions, but they lacked experience. Tasks, activities, or behaviors that are not performed on a routine basis cannot be readily performed during an emergency. The emergency procedures that are required during crises are an extension of basic daily procedures that are performed by emergency medical staff and first responders, such as fire fighters, emergency medical technicians, or police officers.

## Results

The principals in disaster response are the local governments. One of the most important roles of central government personnel is to resolve impediments faced by the local government. To identify these impediments, dispatch of central government employees was extremely useful. The delegation of the C5 section was dispatched to Fukushima for collecting precise medico-social information; this became the catalyst for responding to the crisis. The central government could then evaluate whether the local response system was working smoothly and when something was less than optimal the central government could step in and offer assistance. Recommendations for future medical transportation from lessons learned following the Fukushima nuclear accident are shown in Table 3.

**Table 3:** Recommendations for future disaster medical transportation

Recommendations	
1. Plan for long-distance evacuation	Medical facilities, including nursing homes, should have a plan for long-distance disaster-specific (over 100 km) evacuation. This plan should be practiced with full-scale exercises and when flaws are found, they should be evaluated and eliminated
2. Securement of transportation measures and designated hospitals	A disaster-specific evacuation plan should include the securement of transportation measures and designated hospitals where patients can be sent
3. Multiple communication measures	Healthcare facilities should have two or three independent communication measures such as a radio, satellite phone, amateur radio, and multi-channel access radio systems
4. Supervision by emergency physicians and disaster specialists	Hospital evacuation in disaster settings should be supervised by emergency physicians and be handled by disaster specialists who are accustomed to patient transportation on a daily basis
5. Dispatch of central governmental persons to the disaster site	Selected members of the central government should not stay in the central office waiting for information from the disaster site, but should go into the disaster site, get precise information, and make use of the information to formulate a governmental response
6. The presence of an emergency physician or disaster researcher in the central government	The presence and availability of an emergency physician or disaster researcher in the central government can greatly contribute to the governmental response, especially for disaster-specific medical transportation

## Footnote

This article presents the authors’ personal opinions as emergency physicians/disaster researchers and is not intended to be the official position of the Japanese government.

## Abbreviations

AMES: Aero-Medical Evacuation Squadron
AP: airport
C5: command, control, communications, computers, and counter-intelligence
DMAT: Disaster Medical Assistance Team
ER-HQ: Emergency Response Headquarter
HQ: headquarters
MHLW: Ministry of Health, Labour and Welfare
SCU: Staging Care Unit
AMES: Aero Medical Evacuation Squadron
JSDF: Japan Self-Defense Force
